# Fault interruption scheme for HVDC systems using GaN-HEMT and VCB

**DOI:** 10.1007/s43236-025-01089-z

**Published:** 2025-06-27

**Authors:** Ali Raza, Muhammad Zeeshan Babar, Muhammad Umair Shahid, Hamid Gulzar, Zahid Gulzar, Saif-Ur Rahman

**Affiliations:** 1 Department of Electrical Engineering, Khwaja Fareed University of Engineering and Information Technology, Rahim Yar Khan, Pakistan; 2 School of Engineering and Physical Sciences, The Heriot-Watt University, Edinburgh, Scotland, UK; 3 Arab Technical Co., Ltd, Jeddah, Saudi Arabia; 4 School of Advanced Technology, Xi’an Jiaotong-Liverpool University, Suzhou, Jiangsu China

**Keywords:** Hybrid DC circuit breaker, Vacuum circuit breaker, MESFET, HEMT

## Abstract

Power electronics switching devices played an important role in high-voltage DC circuit breaker development. Timely isolation of faulty portions of an HVDC transmission line from a healthy system is a basic requirement for a fault interruption. In this scenario, the integration of hybrid DC circuit breakers (HDCCBs) with wideband-gap semiconductor devices enables the effective management of high power, currents, and voltages. The SiC-MESFET and the GaN-HEMT are commonly used wideband-gap-based semiconductor devices. This paper introduces a fault interruption scheme for HVDC power systems, featuring the advancement of a hybrid DC circuit breaker. The proposed HDCCB design consists of two parts, one part is based on a VCB as a mechanical circuit breaker, and the second part involves electronic switches for fault interruption. The electronic switches are designed through the combination of GaN and HEMT to achieve fast switching to achieve rapid interruption of fault current. The system model is implemented through a Simulink model to perform a comparative analysis between the presented and existing protection topologies. Current commutation is achieved through the attainment of artificial zero current crossing to interrupt the DC fault. GaN-HEMT emerges as a more reliable and fast switching element compared to other electronic switches like Sic-MESFET as validated by the presented simulative results. The presented model shows better fault-clearing times of 2.2 ms and 2 ms for experimental parameters of (500 kV and 9kA) and (100 kV and 10kA), respectively. This fault-clearing time shows an improvement of 52.38% and 50% compared to the SiC-MESFET-based electronic switches used by the existing mechanisms. The outcomes of the proposed design are evaluated in terms of fault current, commutated current, and voltage across the commutated capacitor.

## Introduction

The rapidly growing demand for electrical energy due to increased industrialization and continuously deteriorating environmental conditions are the major sources of emphasis on RES-based electric power generation. The expansion of HVDC systems to accommodate large-scale implementation of RESs requires electric power to be transmitted over long distances with focus on economical dispatch while incurring fewer transmission losses and improved load flow stability [1, 2]. The main issue when it comes to the implementation of HVDC systems is the fast rise in the fault current due to low impedance compared to HVAC systems, which results in the need for a very fast protection system. Another significant challenge associated with HVDC systems is the lack of inherent zero crossing. Zero crossing serves as the primary function in the operation of a circuit breaker. The circuit breaker plays a pivotal role in safeguarding systems, particularly in managing the specific peak of fault current in HVDC systems, necessitating a dependable design for HVDC circuit breakers [3]. The sustainability of the power network is a primary requisite to ensure the reliable operation of the system.

HVDC systems lack dedicated protection systems and required the development of a hybridized approach to ensure system reliability. In this context, different configurations for DC circuit breakers have been proposed, encompassing mechanical circuit breakers (MCBs), electronic switch circuit breakers, and hybrid circuit breakers that amalgamate mechanical and electronic elements [4]. Each variant of DC circuit breaker presents its own set of pros and cons. The present study introduces a blueprint for a hybrid DC circuit breaker employing GaN-HEMT technology to identify and halt fault currents within HVDC systems.

Modern power systems are designed through a combination of RES and conventional energy sources emphasizing properly designed protection systems with incorporation of HEMT devices. The power transmission in the system depends on both HVAC and HVDC systems. The fault handling in HVAC and HVDC is critical in modern power systems to ensure the secure and dependable operation in hybrid power systems [5, 6]. Hybrid power systems require a sustainable integration of power generation resources to support HVDC topologies for the long-distance transmission of generated power.

The MHVDC system adopts point-to-point power network connectivity for three or more networks. The security of the MHVDC system is a major concern. Research continues to make systems less fault prone with improvements in fault detection, isolation, and restoration process [7, 8]. There are the following points for designing reliable protection DC circuit breakers. 1) The interruption time of the DC fault current is the main criteria in the design of a circuit breaker, which should not be more than 5 ms [9]. 2) The maximum current interruption capability of HVDC CB and to protect the system from the arcing process should be kept in mind. 3) Switching losses are also a main factor. These occur when switching is performed from conduction to interruption states and vice versa. In this state, notable voltages are present across the terminals and high current flows. 4) The rising rates of fault current and harmonics are produced as a result of the delay in interruption/reclosing processes, which disturbs the stability of HVDC systems [10].

In this paper, data from the HVDC circuit breaker study in [11], which is a part of the "National High Technology Research and Development Program of China," are utilized. A comparative analysis with experimental-based research from published papers further validates the model. This work also builds upon and advances the previous simulation-based research, and refines the hybrid DC circuit breaker model.

Fault interruption in advanced HVDC systems requires a solution that is fast, reliable, cost-effective, efficient, and easy to maintain. This research presents methodology using a DC-HCB design that leverages high-electron-mobility transistors (HEMTs) to achieve an exceptionally fast commutation process. Unlike existing solutions, this method minimizes losses and enhances system reliability through an innovative hybrid configuration. The design principles, operational methodology, and unique contributions are elaborated in detail after the related works section, showcasing its potential to address key limitations in current HVDC fault interruption systems.

- a) Research contributions

The principal contributions of this study are outlined as follows.

- Development of a rapid and effective fault interruption process for HVDC systems using a hybrid DC circuit breaker based on a GaN-HEMT and a vacuum circuit breaker (VCB).
- Verification of the efficiency of the proposed design through Simulink-based MATLAB simulations conducted at elevated voltages and currents applicable to HVDC systems.

- b) Paper outline

The remainder of this paper is structured as follows. Section 2 presents the latest advancements in related research. Section 3 outlines the design and operational principles of the proposed hybrid DC circuit breaker. Section 4 presents the results obtained through simulation. Finally, Sect. 5 concludes the paper.

## Related works

Initial attempts at developing HVDC-CBs relied on conventional technologies, such as vacuum circuit breakers (VCBs) and SF6 gas interrupting units. However, these solutions were found to be lacking in speed when it came to fault interruption, leading to heightened economic risks, particularly in instances of short-circuit faults [12]. As a result, researchers began investigating hybrid approaches that combined mechanical breakers with solid-state devices to improve interruption speeds. While traditional MCBs are proficient in current handling, solid-state circuit breakers (SSCBs) demonstrate effectiveness in high-speed, arc-less interruption. Hybrid circuit breakers (HCBs), which merge MCBs with SSCBs, offer a well-rounded combination of their individual strengths [13].

In the early stage of HVDC implementation, traditionally available protection mechanisms, such as mechanical CBs, were used. However, due to inherent complexities involved with the use of power electronic converters, researchers had to design protection equipment having the capability of fast switching. Numerous studies have delved into fault interruption systems incorporating hybrid HVDC circuit breakers, representing the forefront of advancement in this field.

Resonant circuits are used at the zero fault current level [14] and they consist of three parts. 1) The first branch contains a vacuum circuit breaker to interrupt the fault. 2) The second branch consists of a series LC resonant circuit to interrupt the fault current. 3) A bank, made up of surge arrestor and varistors, is used to absorb the energy that dissipated during the interruption process [15]. The types of resonant circuits and their attributes with respect to applications are briefly discussed below.

There are two types of resonant circuits: passive resonant circuits and active resonant circuits. A passive resonant circuit contains an inductor and a capacitor in series parallel with an interrupter such as SF6 [16]. Large oscillations are created in this process and the system may lose its stability. The capacitor starts to charge until the MOV, which controls the fault current and voltage, comes to a certain level. The active resonant circuit in a DC CB is used when the fault is beyond the stability limits. In this technology, a pre-charged capacitor is used to interrupt the fault current [17]. Advancements in breaker operational topologies have motivated power system designers to incorporate modern semiconductors with fast switching capabilities.

The high fault current made it necessary for system planners to install protection equipment with very fast switching speeds to minimize the impact of fault currents like solid-state breakers (SSBs) that are much faster than MCBs. They contain switches such as MOSFETs and IGBTs to interrupt fault current. They can interrupt fault current without zero crossing [18, 19]. In solid-state breakers, bidirectional switching is performed where a series of IGBTs is used to interrupt fault current. There are two branches in a solid-state breaker. The first branch consists of high-voltage surge arresters to protect the switches from dissipated heat during the interruption process. The rating of the solid-state breaker depends on the number of switches connected in the breaker. Under typical operation, current passes through the switches. However, when a fault arises, the switches disconnect the power supply, redirecting current through the MOV in a parallel branch. This action helps restrict the voltage across the switches. Heat losses incurred during the switching process in a solid-state circuit breaker can damage the system and disrupt the stability of the HVDC system [20]. Due to this drawback, SSBs are less reliable compared to MCBs, and required mechanisms to overcome the weaknesses in MCBs and SSBs.

One such approach was to design hybrid circuit breakers (HCBs) by combining the required characteristics of MCBs and SSBs. This type of approach results in a much faster response, a larger current breaking capacity, and reduced energy loses [21, 22]. Mechanical circuit breakers are too slow in their responses but are less expensive than more advanced types. In contrast, the solid circuit breaker responds much faster during the interruption process but the complicated system for handling the losses due to switching make them more expensive. Besides the high fault currents, HVDC circuit breakers also need to tolerate transient voltages emerging in fault conditions.

It should be noted that a perfect DC circuit breaker can withstand transient recovery voltage during the fault interruption process [23]. In mechanical circuit breakers, the triggering gap with a pulse transformer is analyzed [24]. Many topologies have been presented for mechanical circuit breaker in the last few decades [25, 26]. In these topologies, attempts have been made to reduce the commutation time and reduce the fault current. In all of these topologies, mechanical isolators were used to eliminate the fault current path and reduce the arcing effect. Due to the slow operation of the mechanical isolators, a larger trigger gap is required to interrupt the arc, since the fault continues to increase rapidly. These features limit the application of mechanical circuit breakers and require the development of topologies having faster switching speeds to enhance the use of modern HVDC power systems.

The requirements of fast switching require the use of advanced semiconductors in protection equipment to rapidly interrupt fault current. Solid circuit breaker semiconductor devices are used to isolate fault current from a healthy power system in [27]. In solid-state circuit breakers, multiple semiconductors switches, such as thyristor, MOSFET, and IGBTs, are used in series to isolate faults in [17]. Current flows through the metal oxide varistors after opening the switch. When fault current is instantaneously interrupted by the semiconductor switches, the voltage increases abruptly. To limit over voltage, surge arrestors are used in parallel with semiconductor switches [28, 29]. In [30], a solid-state breaker surge arrestor is connected in series with a shunt capacitor. In another solid-state DC CB topology, two joint inductors are used in place of the surge arrestor. A DC shipboard prototype was designed in [22]. The authors also discussed the protection method and its validity testing for DC circuit breakers. Despite the fast-switching capability of SSBs, they are not very reliable due to the heating issue of the ICs used in them. Overheating makes their operation less effective. Thus, more sustainable components need to be integrated with SSBs.

Hybrid DC circuit breakers are a combination of a mechanical circuit breaker and semiconductor switches. In [31], the medium voltage-level application of a hybrid DC circuit breaker was discussed. In [32], the combination of a VI and a silicon carbide solid-state switch was analyzed. A hybrid DC circuit breaker at 10 kV is designed. The major drawback in this prototype is the chance of commutation process failure at high-voltage levels. For higher voltage applications, hundreds of IGBTs are connected in series to manage the transient recovery voltage. In [23], a zero current crossing method was presented with an inductor. In this topology, fault current is interrupted and the requirement of large surge arrestor. A prototype is designed at 4.26 kV, where a thyristor, a capacitor, and a surge arrestor are used [33].

## System model

This section presents the system model for the proposed HVDC fault interruption scheme, covering aspects of both the mechanical circuit breaker and the semiconductor-based circuit breaker. The proposed design incorporates a vacuum circuit breaker (VCB) as the mechanical circuit breaker, and utilizes a high-electron-mobility transistor (GaN-HEMT) as the solid-state device, forming a hybrid switching mechanism. The analysis in this work includes a performance comparison between GaN-HEMT and MESFET, aiming to determine the optimal choice for the proposed hybrid DC breaker (HCB). First, fault interruption management using VCB is explained, followed by a detailed account of the HEMT component, which enables a thorough grasp of the proposed hybrid DC breaker.

- a) Interrupting high-voltage DC with vacuum circuit breakers

**Fig. 1:**
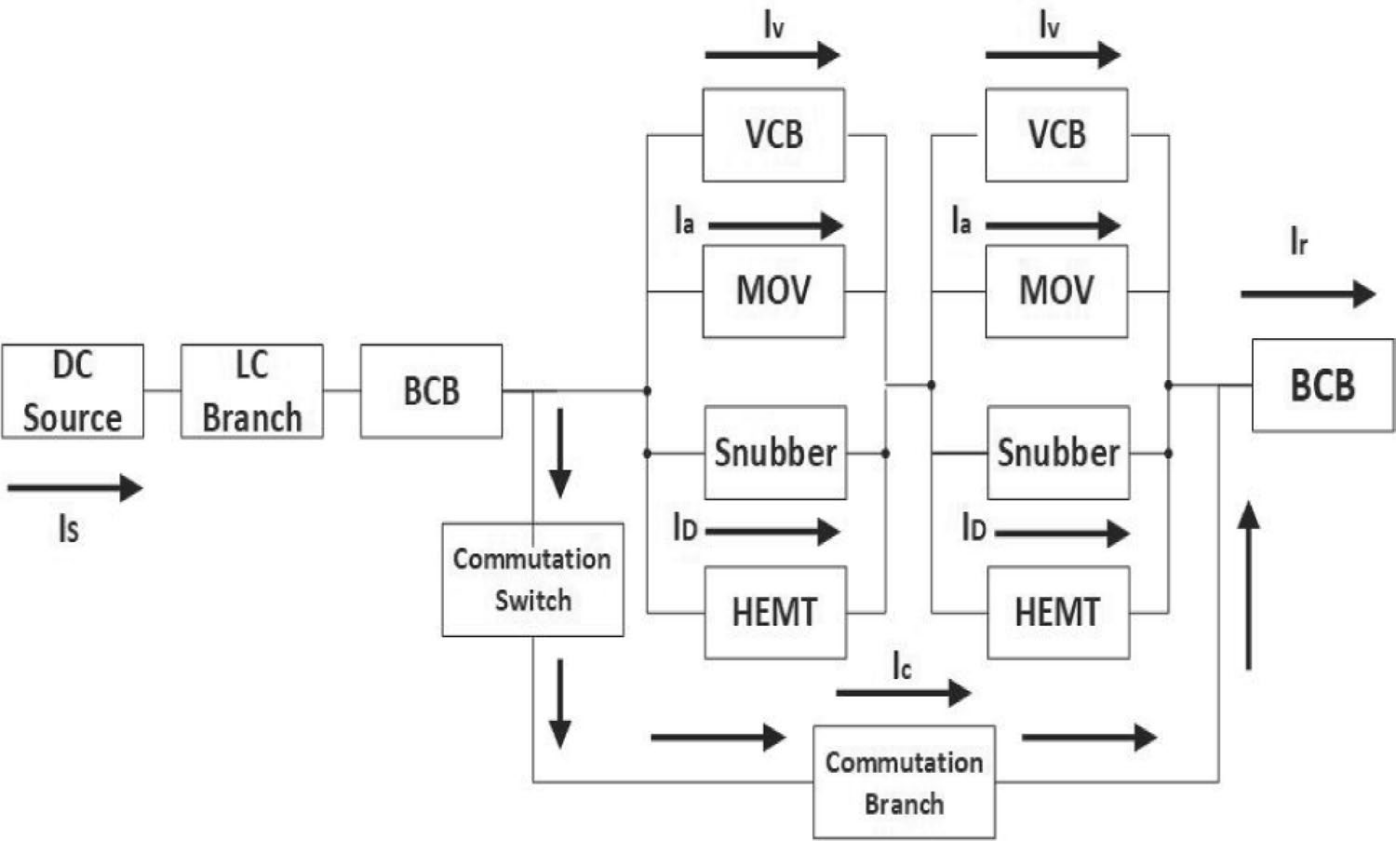
Block diagram of hybrid DC interruption with MESFETs

A block diagram illustrating the fault interruption process using a VCB is depicted in Fig. 1. In this configuration, three branches are evident: the VCB itself, metal oxide varistors (MOVs), and the commutation branch, which includes a pre-charged capacitor *C* linked in series with an inductor *L*. When a fault occurs, as soon as the fault current impulse reaches a secure level, the contacts of the VCB separate, causing an arc to form between them. Following this, the switch *S* in the commutation branch is activated, allowing the pre-charged commutation capacitor and inductor to produce high-frequency oscillations. These oscillations blend with the VCB current, establishing an artificial zero-crossing point, which aids in extinguishing the arc initiated by the VCB contacts. The strong capability of the VCB to extinguish arcs is credited to its excellent insulation.

**Fig. 2:**
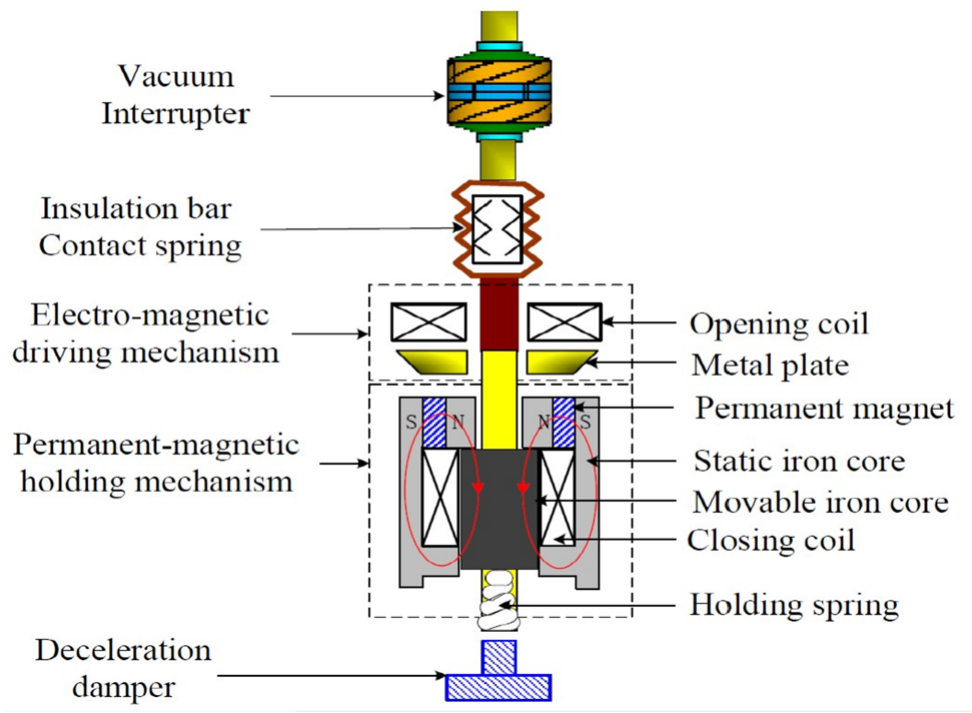
Vacuum circuit breaker (VCB) structure diagram

The functionality of the VCB relies on electromagnetic repulsion (EMR) and permanent magnet (PM) mechanisms, as depicted in Fig. 2. During a fault event, the ERM component ensures swift responsiveness, while the PM system mitigates the mechanical impact induced by the ERM. Further details on this process can be found in [6].

In this study, for streamlined operation, two small-gap VCBs are employed instead of a single long-gap VCB. This decision reduces the VCB response time during fault interruption, thanks to the smaller moving parts and the axial dimensions. Additionally, the combination of VCBs reduces the transient recovery voltage (TRV) associated with interruption events. TRV, which manifests as voltage across the contact gap during interruptions, is influenced by the reactive elements present on both the line and load sides of the VCB [34]. The performance of a VCB usually depends on variables like the insulation capacity between contacts when opening, and its ability to extinguish high-frequency currents precisely at zero crossings.

### High electron mobility transistor (HEMT)

**Fig. 3:**
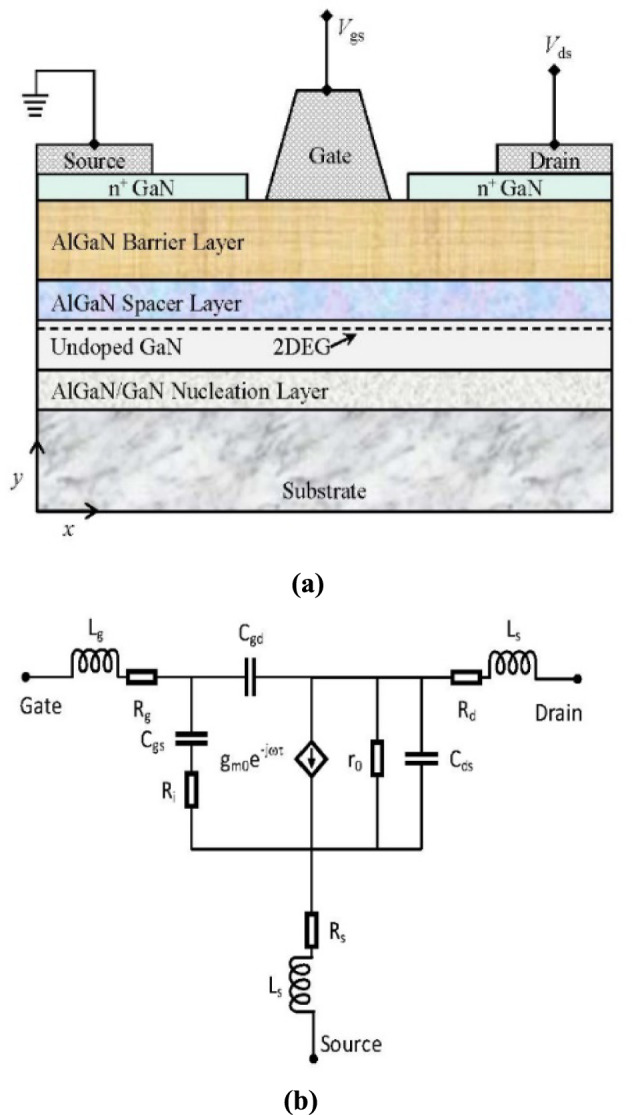
AlGaN/GaN-HEMT: **a** detailed layer structure [34]; **b** AC equivalent structure [34]

Compound semiconductor devices are the main building blocks in the current electronic era that have led to more cutting-edge electronics applications. The detailed structure and equivalent model of AlGaN/GaN-HEMT are shown in Fig. 3. Wide band-gap $(E_{g} > 3{\text{ eV}})$-based semiconductors can retain their fundamental characteristics at a higher temperature (500 °C). Therefore, they are preferred for both high-power and microwave devices for both room-temperature and harsh environmental conditions. The effectiveness of a semiconductor device for a particular application is contingent upon its ability to withstand breakdown under an applied electric field, its carrier mobility, thermal conductivity, and intrinsic carrier concentration relative to temperature. GaN material emerges as a competitive alternative that meet these criterions, making it a strong contender owing to its wide bandgap in the range of 3.39 eV [35].

**Fig. 4:**
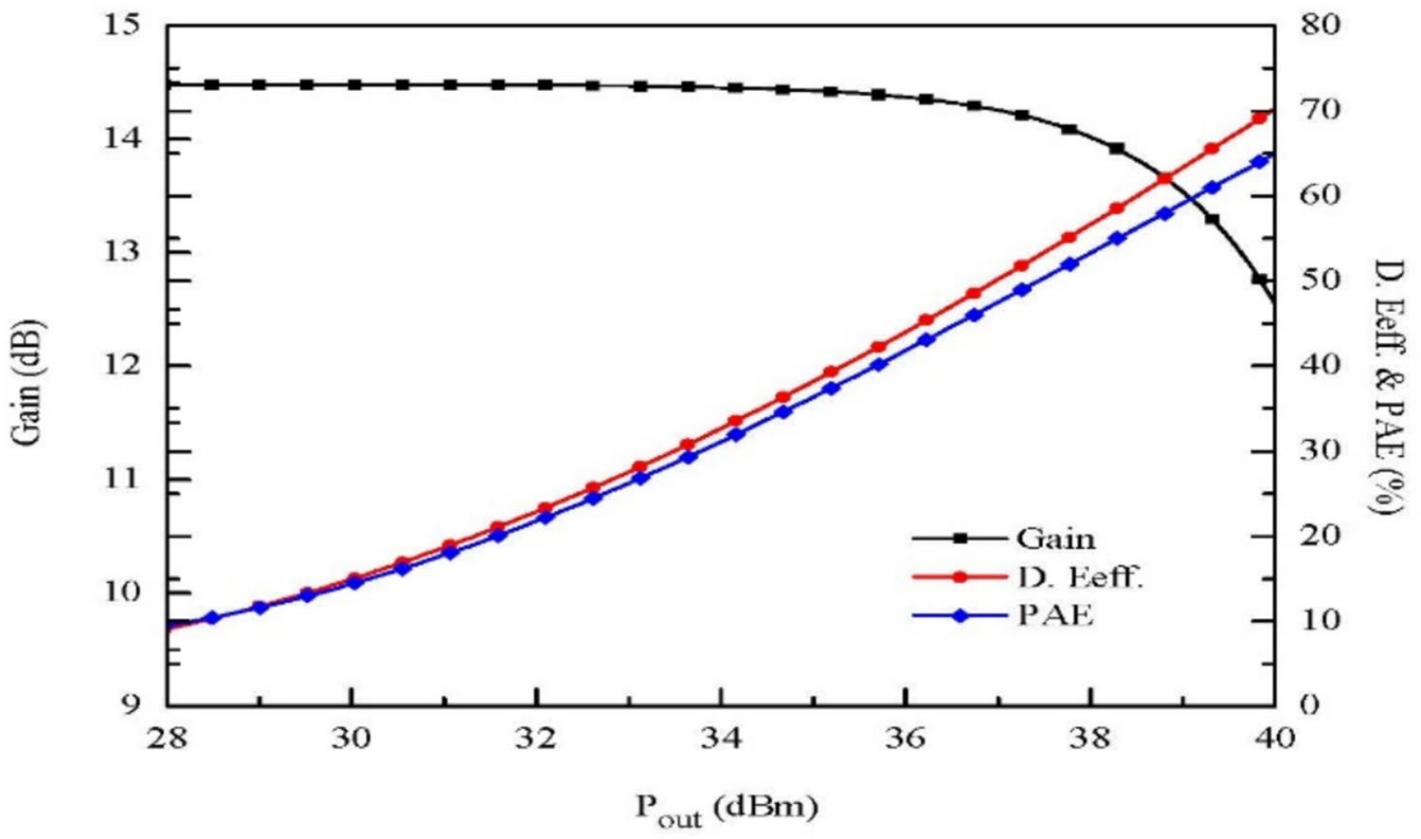
Different types of commonly used power electronics transistors expressed in terms of the threshold voltage (${V}_{br}$) and the unity gain frequency (${f}_{T}$) [36]

Great progress has been made in GaN HEMTs from high-frequency and high-power perspective. They can operate at elevated temperatures, up to 500$^\circ{\rm C}$, due to their high breakdown field of 3.3 MV/cm, which is basic requirement for power transistors during high bias operation. Figure 4 shows the various types of commonly used power electronics transistors, expressed in terms of the threshold voltage (${V}_{br}$) and unity gain frequency (${f}_{T}$).

**Fig. 5:**
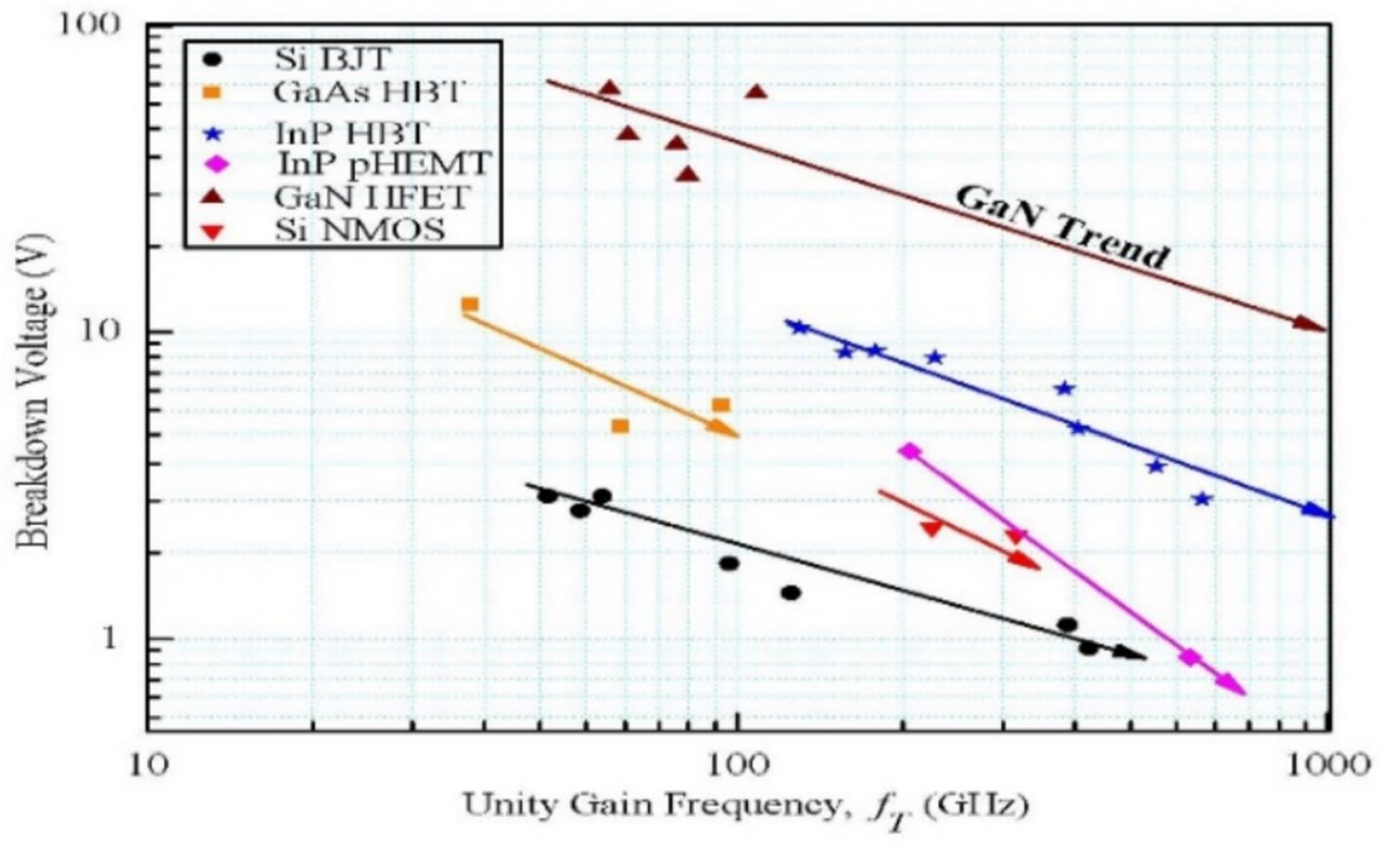
GaN-HEMT device performance analysis at ${V}_{ds}=28V$ for 6 GHz [36]

For a 20A device, high mobility combined with a high charge concentration ${n}_{s}$ in 2DEG gives low resistance up to ${R}_{on}<100 m\Omega$, which directly effects the switching losses of the device. In addition, AlGaN/GaN-based HEMTs have ${Q}_{g}$ and ${R}_{on}$ as given by (${Q}_{g}\times {R}_{on}<1 nC$). The value is lower than the Schottky barrier gate value and fewer switching losses are encountered, making it suitable for high-power adaptation in the switching mode [37, 38]. Figure 5 shows the performance analysis of an HEMT device with drain efficiency, gain, and power added efficiency (PAE) vs Pout at ${V}_{ds}=28 V$ for 6 GHz.

- b) Proposed hybrid DC circuit breaker

**Fig. 6:**
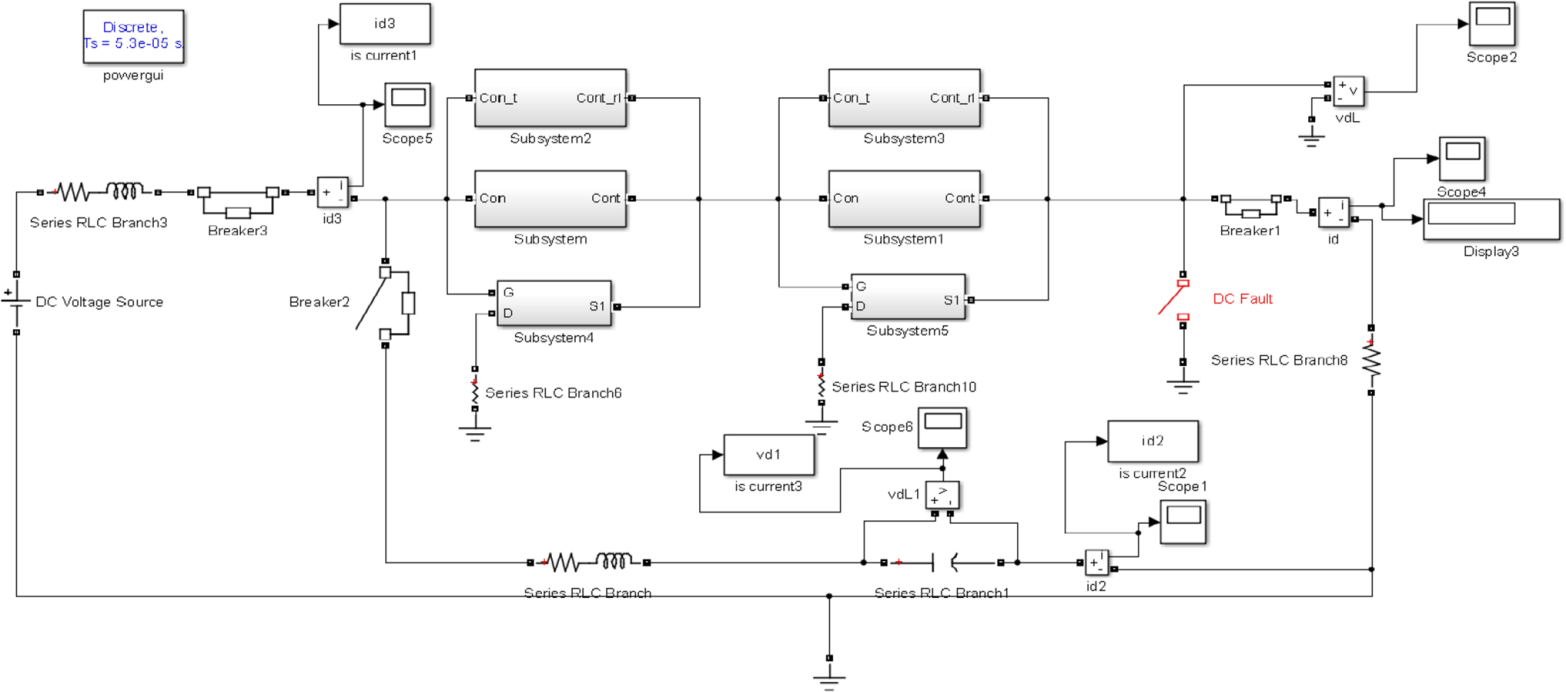
Simulation model of hybrid DC interruption with GaN-HEMT

**Fig. 7:**
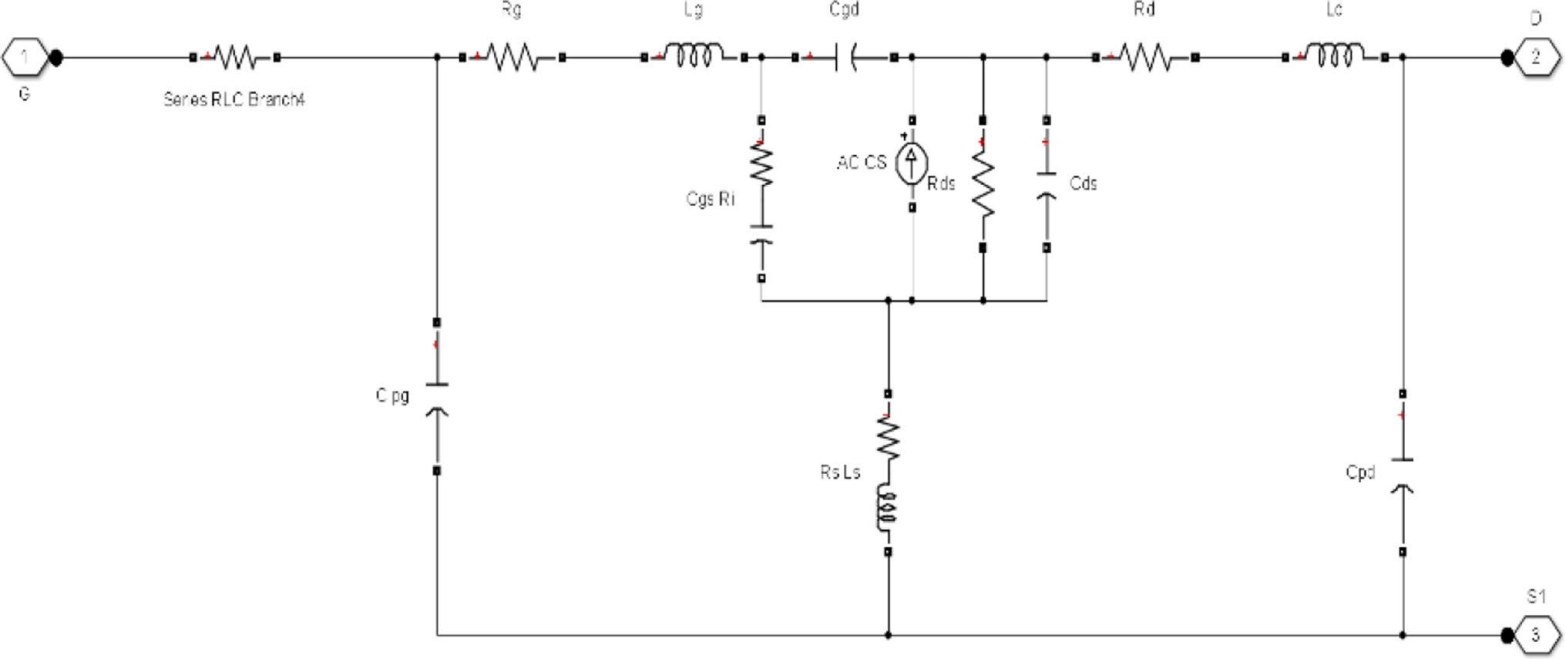
HEMT simulation diagram

The proposed model is divided into multiple series modules, because relatively smaller mechanical circuit breakers are easy to handle and suitable for commutation processes. Each module contains four parallel branches such as VCB, metal oxide varistor (MOV), commutation branch, and GaN-HEMT. The schematic diagram of the proposed model is shown in Fig. 6, where two submodules are connected in series and the VCB is controlled by GaN-HEMT. The detailed working principle of the VCB is described in [38]. When a fault occurs in the system, the GaN-HEMT equivalent model shown in Fig. 7 operates and produces a magnetic field around it through a pulse current *i*_*1*_. In addition, eddy current is produced by the magnetic field. Repulsive force is generated between the metal plate and the coil, which starts the rapid movement of these plates. In this topology, the capacitor *C* is charged through the *RLC* circuit, and the diode *D* is used to discontinue charging, once *C* is fully charged. In this configuration, the circuit operates as an *RL* resonance circuit. The simulation parameters are given in Table 1:

**Table 1:** Simulation parameters

Parameters	Values
Source voltage	80 kV
Resistance	0.79
Inductance	23mH
Output resistance	100Ω
Charging resistance	3000Ω
Surge arrestor (MOV)	80 kV
Voltage across commutation capacitance	80 kV
Commutation frequency	4 kHz
Maximum current	4.2KA
Commutation capacitance	6e-6uF
Commutation inductance	39uH
System Current in normal mode	1KA
Counter current after fault	10 KA injected at 3 ms
*GaN-HEMT parameters*	
Gate length Lg	0.5um
Width W	400um
Battery layer doping density	6 * 10^15 cm^3
Input resistance Ri	1.01Ω
Source resistance Rs	260.6Ω
Gate resistance Rg	5Ω
Drain resistance	0.143Ω
Voltage across commutation capacitance	80 kV
Commutation frequency	4 kHz
Maximum current	4.2KA
System current in normal mode	1KA
Counter current after fault	10KA injected at 3 ms

The detailed interruption process with respect to time is described as follows.

- *T0:* In the normal working state, the capacitor is fully charged and stable.
- *T1:* The fault current swiftly flows through the system upon the occurrence of a fault. The HEMT detects the fault current and gets triggered. This creates a pulse of current, and the ERM process is initiated. The VCB contacts open during the ERM process and arcs appear between the contacts of the VCB.
- *T2:* The commutation switch monitors the TRV. When it reaches a predetermined threshold, the switch closes, initiating the commutation process through the commutation current.
- *T2 –T3:* Current shifts to commutation branch after closing the commutation switch. Meanwhile, the pre-charged capacitors begin to discharge.
- *T3:* When the current is switched to the commutation branch, the initial commutation procedure is finished. The arc extinguishing process begins when the VCB opens, the TRV reaches a safe limit, and the snubber circuit starts working.
- The snubber circuit is responsible for two important points:
- Initially, the substantial capacitance is uniformly dispersed within the snubber branch throughout the TRV process.
- Then the high oscillating frequency of the TRV is controlled by the RC circuit.

- *T3 – T4:* High energy emerges upon extinguishing the arc due to the deliberate creation of zero current. The inductor, integrated in series with the surge arrestor, dissipates this energy. Furthermore, the second commutation initiates when the current transitions from the commutation branch to the arrestor branch. At this point, the voltage of the pre-charged commutation capacitor diminishes toward zero.
- *T4:* Once the total current is directed to the surge arrestor branch, the second phase of commutation is finalized.
- *T5:* The total equivalent current passes through the varistor and the commutation circuit, which includes the charging capacitor.

## Simulation model, results, and discussion

**Fig. 8:**
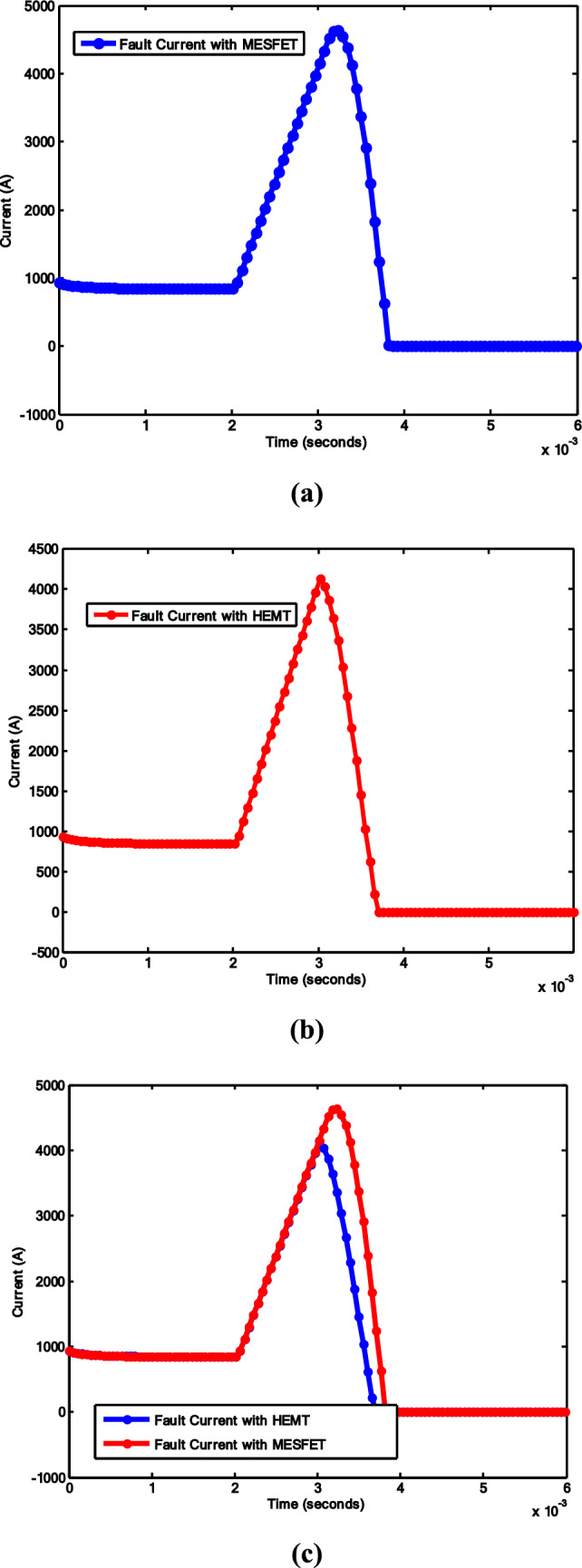
System current results: **a** HCB with MESFET; **b** HCB with HEMT; **c** comparison between **a** and **b**

This section describes the proposed model for the fault current interruption process, which is implemented in MATLAB Simulink. The simulation is segmented into the main circuit breaker utilizing VCB, the HDCCB utilizing HEMT with VCB, and the intricate MCB model, which is outlined in [39]. The suggested model of the HDCCB utilizing GaN-HEMT (as shown in Fig. 3) is used in Figs. 6 and 7. Additionally, Table 1 presents the simulation parameters, while Figs. 7 and 8 depict the corresponding outcomes. The system, which is constructed with two VCB modules connected serially, assumes the source voltage on the left side and the fault occurrence on the right side (as depicted in Fig. 6).

Figure 8 illustrates the normal condition with a 1kA current flow and a fault transpiring at 2 ms in both scenarios: Fig. 8a HCB with MESFET and VCB, and Fig. 8b HCB with GaN-HEMT and VCB. Concurrent with the fault occurrence, the current begins to rise, peaking at 4.2kA, thereby initiating the opening of the VCB contacts and the closure of the commutation switch *S*.

Figure 8 illustrates that arc quenching is finalized within 3.8 ms. In Fig. 8a, the time taken is 1.9 ms to reach zero crossing. The fault interruption time of the proposed circuit breaker is 1 ms. Two backup CBs (BCBs) are linked at both ends of the system. The primary function of these BCBs is to restrict residual current through post-arc quenching. Figure 8c presents a comparison of scenarios, showcasing that HEMT-based HCBs perform better, since they settle to zero current earlier.

**Fig. 9:**
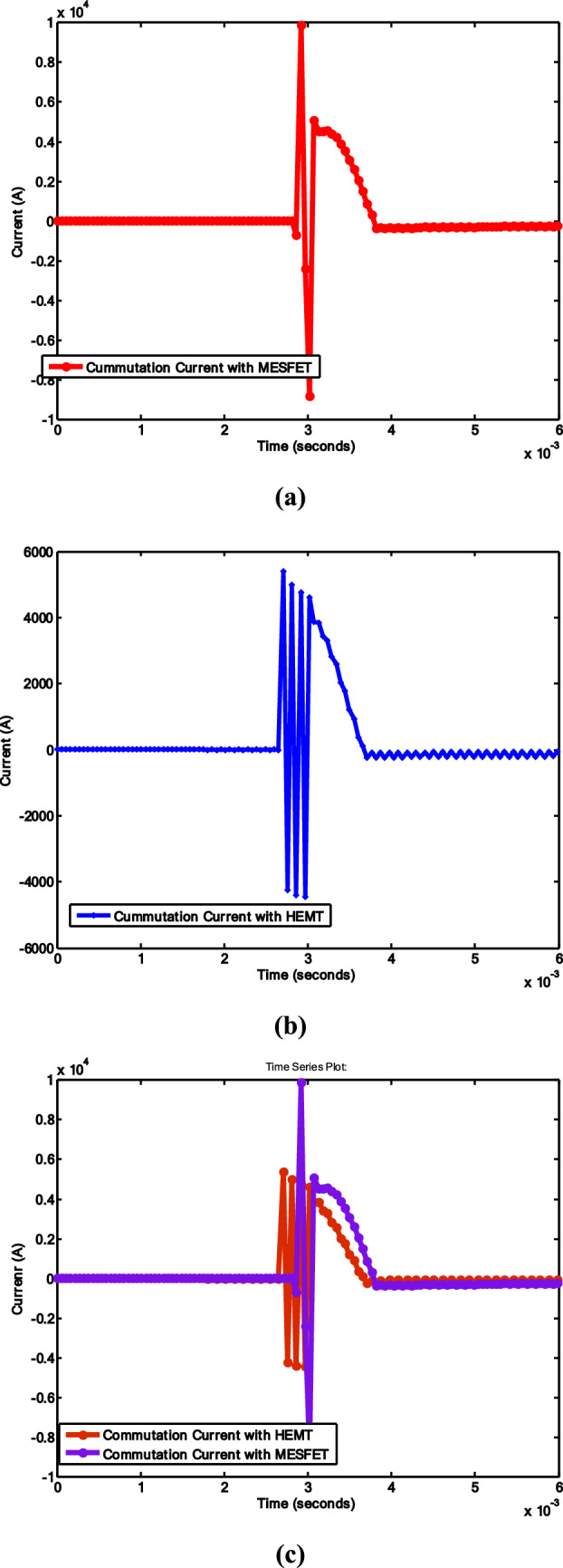
Commutation current results: **a** HCB with MESFET; **b** HCB with HEMT; **c** comparison between **a** and **b**

Figure 9a and b shows results for the flow of high currents and simultaneous closure of the commutation switch during the occurrence of high oscillating currents while considering HCBs using MESFET and HEMT, respectively.

**Fig. 10:**
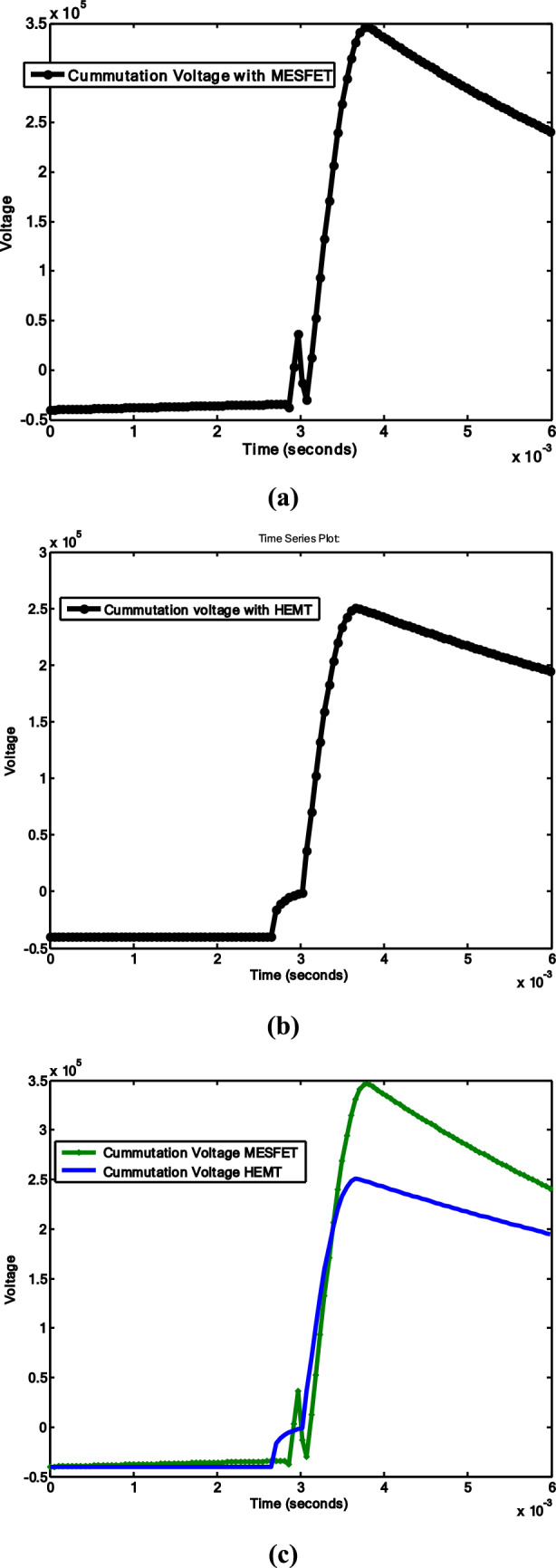
Commutation voltage results: **a** HCB with MESFET; **b** HCB with HEMT; **c** comparison between **a** and **b**

The impacts of SiC-MESFET and GaN-HEMT with the VCB on the commutation voltage are shown in Fig. 10a and b, respectively. The commutation current decreases to 5.6kA in comparison to the 10kA of the MESFET. The size of the surge arrester is reduced because of the low peak of commutation current.

The commutation voltage appears across the capacitor, as shown in Fig. 10. The results show the process when the commutation switch closes. The commutation process initiates upon the closure of the commutation switch, prompting the discharge of the capacitor. The size of the surge arrester is reduced due to the low peak of the commutation current. The heat dissipation in the circuit breaker is reduced and the power loss is reduced. The dielectric strength of the VCB stands easily on the low peaks of current. The reduction in the size of the surge arrester leads the low cost of the overall system design.

**Fig. 11:**
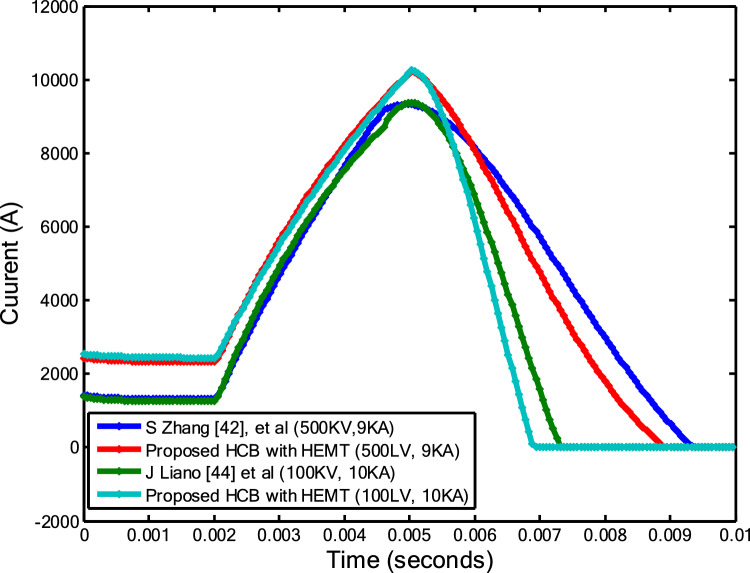
Comparing the fault current breaking times between the proposed design and existing works [39, 40]

Figure 11 highlights the importance of the improved performance of the HCB design employing SiC-MESFETs, especially concerning the fault current breaking time.

Table 2 is based on two setups: one at 200 kV with a peak current of 10kA; and the other at 500 kV with a peak current of 9kA.

**Table 2:** Simulation results

	S Zhang, et al. [40]	Proposed HCB with GAN-HEMT	J. Lianoi [41],	Proposed HCB with GAN-HEMT
Fault clearance time	500kV and 9kA		100kV and 10kA	
	4.2ms	2.2ms	4ms	2ms

The presented outcomes showcase the superior performance of the presented protection topology, with its reduced time in removing the fault current.

## Conclusion

This paper presented an improved model for fault interruption in HVDC systems, utilizing a hybrid circuit breaker that combines GaN-HEMT and VCB technologies. This is accomplished by connecting multiple series modules to operate at low-voltage levels. The presented protection topology achieves a reduction in commutation current compared to MESFET-based systems. The primary aim of this study is to minimize fault clearance time, showcasing superior performance compared to the existing methods, as depicted in the presented results. Numerous benefits arise from the decreased commutation current, such as a smaller surge arrester footprint, a reduced heat dissipation in the circuit breaker, lower power losses, and improved dielectric strength of the VCB, enabling it to withstand low current peaks. The smaller surge arrester size contributes to a more cost-effective overall system design.

## Data Availability

Data sharing does not apply to this article as no datasets were generated or analyzed during the current study.
